# Introduction to the Special Issue on “Keratinocyte Carcinomas: Biology and Evolving Non-Invasive Management Paradigms”

**DOI:** 10.3390/cancers15082325

**Published:** 2023-04-17

**Authors:** Salvador González, Melissa Gill, Ángeles Juarranz

**Affiliations:** 1 Department of Medicine and Medical Specialties, Alcalá de Henares University, 28805 Madrid, Spain; 2 Department of Clinical Pathology and Cancer Diagnostics, Karolinska University Hospital Solna, 17176 Stockholm, Sweden; 3 Department of Pathology, SUNY Downstate Health Sciences University, Brooklyn, NY 11203, USA; 4 Department of Biology, Faculty of Sciences, Universidad Autónoma de Madrid, 28049 Madrid, Spain

## 1. Introduction 

Keratinocyte carcinomas (KCs) are the most prevalent form of cancer worldwide, and their incidence is rising dramatically, with an increasing trend in recent years. Among the types of KCs, basal cell carcinoma (BCC) has the highest prevalence, and squamous cell carcinoma (SCC) is less common, although it has a higher propensity to metastasize, accounting for the majority of KCs-related deaths. High-risk KCs that present on the face can cause morbidity and mortality and incur significant costs associated with treatment. Non-invasive diagnostic tools, such as dermoscopy, reflectance confocal microscopy (RCM), and line-field optical coherence tomography (LC-OCT), among others, have been introduced into clinical practice to facilitate better management of KCs. Established non-surgical treatment options, such as photodynamic therapy (PDT) and topical imiquimod, have the additional advantage of providing both field treatment and optimal aesthetic results. 

## 2. Clinical Snippets

Gallego-Rentero, M. et al. [1] conducted research focused on the role of the tumor microenvironment (TIME) in the resistance to Photodynamic therapy (PDT). In particular, the authors evaluated the role of TGFβ1 produced by cancer-associated fibroblasts (CAFs). The authors isolated fibroblasts from tumor biopsies from patients and, in vitro, characterized the cells and measured the levels of TGFβ1 secreted by CAFs. The novelty of this work is that the authors demonstrate that TGFβ1 is responsible for SCC resistance to PDT and, therefore, propose the level of this cytokine may be used as an indicator of resistance to PDT.

Ruini, C. et al. [2] performed a study aimed to evaluate the application of a machine learning (ML) algorithm for detection of SCC in digitally stained images obtained by ex vivo confocal laser scanning microscopy (CLSM). Thirty-four freshly excised tissue samples (22 invasive SCC and 12 tumor-free controls) from 29 patients were examined by experts using digitally stained ex vivo CLSM and diagnosis was confirmed by conventional histopathology. CLSM images were annotated by experts and split into smaller submosaics, which were then used to train and test a MobileNet convolutional neural network (CNN). Compared to expert examination, the overall sensitivity and specificity of the CNN in detecting SCC and tumor-free skin in the digitally stained ex vivo CLSM images was 0.76 and 0.91, respectively, confirming that this ML tool can detect SCC regions and distinguish them from tumor-free areas with good sensitivity and specificity. These findings suggest that integration of artificial intelligence tools may optimize the diagnostic process by increasing accuracy and reducing the time required for CLSM image interpretation. Futher studies are needed to develop standardized acquisition methods and increase the sensitivity and specificity of ML algorithms.

Curiel-Lewandrowski, C. et al. [3] examined RCM criteria for actinic keratosis and determined that atypical honeycomb pattern, hyperkeratosis, stratum corneum disruption, and disarranged epidermal pattern enabled the most accurate distinction from surrounding photodamaged skin and displayed the most significant change following therapy (cryotherapy or PDT) with a good interobserver agreement. In this study with limited follow-up, clinical evaluations also performed well in assessing the response to therapy. However, interestingly, the authors also report that RCM detected worsening of parameters at 6 months post-therapy compatible with early recurrence, which was not detected by a clinical exam. These findings support the hypothesis that RCM assessment is more sensitive than clinical examination allowing for detection of early (subclinical) relapse. By enabling non-invasive dermatopathology, RCM may increase the accuracy of outcome assessment and allow for insight into the mechanisms behind treatment response. However, due to the inherent limited imaging depth of RCM, only features present in the superficial skin can be evaluated, which may not provide a complete picture of the underlying biological processes.

Verzì, A.E. et al. [4] conducted a study to assess the advantages of LC-OCT vs. clinical evaluation for monitoring superficial BCC. For this purpose, clinical and LC-OCT evaluations of 20 superficial BCCs from 12 patients treated with imiquimod 5% cream were performed at baseline and 4 weeks after the end of the treatment. Four weeks post conclusion of imiquimod treatment, 13 lesions showed complete clinical and LC-OCT response, 4 lesions showed partial clinical and LC-OCT response, and 3 lesions showed discordant results. For all 3 discordant lesions, clinical evaluation showed complete response, while LC-OCT revealed the presence of lobulated structures connected to the epidermis, a major sign of residual BCC. Despite the limitations of this study (small sample size, short follow-up period, etc.), these results suggest that LC-OCT could enhance evaluation of BCC response to non-invasive treatments, which is important since these cutaneous tumors constitute the most common type of skin cancer and, when undertreated, can be highly mutilating leading to significant morbidity.

Giovannacci, I. et al. [5] assessed whether specific histological features may account for changes in autofluorescence (AF). In this ex vivo study, AF spectroscopy followed by histopathology was performed on lesional and surrounding non-lesional tissue from 98 surgical excisions of suspected NMSC. Statistical analysis was performed to assess for correlations between autofluorescence intensity ratio (AFIR) and histological diagnosis including subtype; presence or absence of elastosis and fibrosis; and grade (0–3) of neovascularization, epithelial thickening, hyperkeratosis, and cellular atypia. All BCCs (59) and 90.4% (47) of squamous precancers and cancers showed a decrease in AF compared to surrounding skin, but no statistical significance was found between mean BCC (4.5) and SCC (4.4) AFIR. While many histological subtypes showed a wide AFIR, outliers included pigmented BCC (mean 12.8, range 9.908–15.73) and keratoacanthoma (mean 1.8, range 1.296–2.240). Using linear regression models, stromal features (fibrosis, elastosis and neovascularization) appear to contribute the most to changes in AF. By contrast grade 3 hyperkeratosis was the only tumor parameter which showed statistically significant correlation with AFIR. The results of this study suggest that AF spectroscopy may help in delineting tumor margins to achieve complete excision in primary biopsies. However, further studies are needed to understand if AF can be used to distinguish keratinocyte carcinomas from scar tissue and benign mimickers, especially those with increased vascularity or fibroplasia.

Oya, K. et al. [6] aimed to determine if the combination of imiquimod (IMQ) and anti-PD1 enhances antitumoral effects and elucidate the underlying antitumor mechanisms of IMQ alone and IMQ-anti-PD1 combination therapy. For this purpose, human tumor cells (B16F10 melanoma, Lewis lung carcinoma (LLC), MB49 bladder cancer, and MC38 colon cancer) were intradermally injected into wild type C57BL/6J mice, and resultant tumors were treated with topical IMQ and intraperitoneal injections of anti-PD1 antibodies. Topical IMQ did not exert significant antitumoral effects against melanoma or LLC cells but significantly inhibited tumor growth of bladder and colon cancer cells by enhancing the immune response. From this, it was concluded that the antitumor effect of topical IMQ depends on an enhanced immunologic response to the tumor since both MB49 and MC38 have been reported to show high immunogenicity, whereas B16F10 and LLC are poorly immunogenic tumors. Results from in vitro assays and in vivo experiments using IFN-γ deficient, Rag1-deficient and homozygous CD19-Cre transgenic mice suggest that imiquimod response is dependent on adaptive immunity and increased production of IFN-γ (specifically increased expression by CD8+ T cells) and raise the possibility that B cells may not play a major role. Interestingly, the study also found that IMQ upregulated PD-L1 expression through an IFN-γ-independent mechanism. Finally, combination therapy resulted in a significant potent antitumor effect compared to either therapy alone. While this may be due to additive effects of IMQ and anti-PD-1 antibody in activating innate and adaptive anti-tumor immunity, a synergistic effect created by anti-PD-1 antibody blockade of IMQ-induced PD-1 expression could play a significant role. In summary, depending on the immunogenicity of the tumor, combination therapy with topical IMQ plus anti-PD-1 antibody may play a promising role in the treatment of primary and secondary cancers of the skin.

Santiago, J.L. et al. [7] developed a preclinical model of skin with cutaneous field cancerization after chronic ultraviolet (UV) B light exposure in immunocompetent SKH1 aged mice. Compared to non-irradiated controls, UV-B irradiated skin demonstrated impairments in trans-epidermal water loss, stratum corneum hydration, and surface pH in a dose-dependent manner. A similar correlation between UVB irradiation and histological changes including epidermal thickening (acanthosis with hypergranulosis); hyperkeratosis; superficial vascular ectasia; increased number of mast cells; increased expression of PCNA, p53, filaggrin, loricrin and involucrin by immunohistochemistry; increased lipid content in the superficial epidermis by Nile red staining; and development of AKs and in situ SCCs were identified. These findings suggest permeability barrier impairment, keratinocyte hyperproliferation and mast cell activity may also be useful targets for the development new therapies to prevent and treat UV-B induced skin cancer.

Ruini, C. et al. [8] aimed to determine if assessment of AK progression risk using the PRO I-III histopathological classification system could be achieved non-invasively using LC-OCT. Vertical LC-OCT images and vertical hematoxylin and eosin-stained routine histopathology sections from 50 histologically confirmed AKs were evaluated, and the highest observed proliferation grade for each was recorded using each modality in a blinded fashion. To assess reproducibility, grading with LC-OCT was performed by 4 experts in noninvasive diagnostics, a pair of dermatologists (observer round) and a dermatopathologist and a dermatologist with extensive dermatopathology training (consensus round). A total of 17 AKs were histologically classified as PRO I, 22 as PRO II, and 11 as PRO III. The LC-OCT PRO grading was in agreement with the histological grading in 75% of lesions, and the interobserver agreement for LC-OCT PRO grading between the observer round and consensus round was 90%. Although larger studies are needed to validate these findings, LC-OCT seems to enable reliable in vivo evaluation of proliferative growth in AKs, thereby allowing for non-invasive identification of AKs with high risk for progression to invasive SCC.

Stanganelli, I. et al. [9] performed a comprehensive review of the diagnosis, treatment, and follow-up of SCCs and proposed updated guidelines. By collating and comparing the myriad complex epidemiologic data, diagnostic tools, prognostic factors, treatment options, and follow-up recommendations, the authors created an indispensable single reference resource. The collated information was then reviewed by a panel of experts, and new consensus recommendations were developed with the aim to achieve a more comprehensive, streamlined multidisciplinary approach to SCC diagnosis and management.

Vlachos, C. et al. [10] compiled data published in the literature on paraneoplastic syndromes (PNS) associated with keratinocyte skin cancer (KSC). They indicate that a prior review publication on this topic was not identified. The authors found a total of 41 reported cases KSC-associated PNS, the most common of which were malignancy associated hypercalcemia (MAH; 78%), anemia (10%), and Bazex syndrome (5%). Most of the PNS (85%) occured in association with SCC, 10% with BCC, and the rest with adnexal tumors. The median patient age at the time of PNS diagnosis was 58 years. Scar or hidradenitis suppurativa, KSC-predisposing conditions, were reported in >70% of the PNS cases. While exisiting literature does not support the concept that KSC can cause PNS, the authors report that the PNS resolved after treatment of the KSC in the majority of cases presented in this review, suggesting otherwise. 

Omland, S.H. et al. [11] reviewed the literature indicating that systemic immunotherapy can be applied for keratinocyte carcinoma (KC), specifically for locally advanced and metastasizing basal and squamous cell carcinoma. In addition, the authors indicate that lasers can activate an immune response in the skin. Moreover, immune activation by lasers combined with immunotherapeutic agents, known as laser immunotherapy, produces not only direct anti-tumor effects but also systemic adaptive immunity that prevents tumor recurrence and regression in distant untreated tumors. Therefore, based on published data, the authors suggest that laser immunotherapy would be an excellent treatment for KCs with therapeutic potential for both local and metastatic disease. 

Pihl, C. et al. [12] reviewed the photoprotective potential of various pharmaceuticals, phytochemicals and vitamins for prevention of KCs. Repurposed pharmaceutical compounds constitute a promising option for systemic photoprotection. Non-steroidal anti-inflammatory drugs (NSAIDs), such as celecoxib, nimesulide, or acetylsalicylic acid, have demonstrated protective potential in clinical trials, reducing KCs and promoting AK regression. Metformin (AMPK activator), resatorvid (selective Toll-like receptor 4 antagonist), Prinaberel (selective oestrogen receptor beta agonist), and carvedilol (β_1_, β_2_ and α_1_-adrenergic receptor antagonst) delay tumor onset and reduce tumor incidence and multiplicity in mice. Bucillamine (a cysteine-derived compound with structural similarities with N-acetylcysteine) is a potent antioxidant that has been reported to reduce proliferation, cell cycle arrest, and apoptosis in vitro. Moreover, non-specific oestrogenic compounds, such as 17β-oestradiol, reduce UV-induced immunosuppression. A second group, plant-derived compounds or phytochemicals, are also promising photoprotectants. Polyphenols are particularly common photoprotective phytochemicals that act as antioxidants. Polyphenols from green tea (epigallocatechin-3-gallate); grapes (proanthocyanidins and resveratrol); cocoa (flavanol); and those present in polypodium leucotomos (a fern), pomegranate, and raspberries have been shown to delay tumor onset and reduce tumor incidence, size, multiplicity, and progression in mice, preventing both development AK and progression to SCC. Finally, the photoprotective properties of vitamins C, D3, and E (α-tocopherol) contribute to delay of tumor onset and reduction of tumor incidence, size, multiplicity, and progression in mice. Vitamin A (retinoids) and B_3_ (nicotinamide) have also been shown to reduce AK occurrence and SCC risk. Despite this evidence, more studies are needed to optimize use and identify the most promising targets for photoprotection.

Romano, A. et al. [13] summarized the state of the art and proposed incorporating non-invasive approaches to aid diagnosis of oral SCC, which is responsible for 90–95% of malignant tumors of the lip and oral cavity and associated with high mortality when advanced in stage. The authors reviewed non-invasive in vivo diagnostic techniques ranging from inexpensive, simple procedures to innovations requiring specialized devices, including vital staining, tissue autofluorescence, narrow-band imaging, high-frequency ultrasound, optical coherence tomography, and confocal microscopy. The authors state that biopsy for histopathological assessment is the gold standard for OSCC diagnosis; however, patient aversion to invasive biopsy and challenges identifying the most representative area to biopsy, necessitating multiple biopsies can lead to delays in diagnosis. The authors propose a 3-step diagnostic process incorporating these non-invasive techniques would improve diagnostic accuracy and thereby decrease the number of unnecessary invasive procedures, resulting in improved patient satisfaction and decreased delay in diagnosis.

Caini, S. et al. [14] conducted a systematic review of the literature and meta-analysis of studies exploring associations between vitamin D and KCs. No significant association between NMSC risk and vitamin D intake (from diet and supplements), vitamin D blood concentration, or polymorphisms of the vitamin D receptor and binding protein genes was identified. The authors conclude that the relation between vitamin D and KCs in humans remains unclear. However, the results of certain studies, such as the reported positive association between BCC and vitamin D intake, are worthy of further investigation.

In this issue, we present relevant findings regarding noninvasive diagnosis and non-surgical treatment of KCs of the skin and oral mucosa and highlight knowledge gaps in KC pathogenesis and management yet to be resolved.

## References

[1] Gallego-Rentero M., Gutiérrez-Pérez M., Fernández-Guarino M., Mascaraque M., Portillo-Esnaola M., Gilaberte Y., Carrasco E., Juarranz Á. (2021). TGFβ1 Secreted by Cancer-Associated Fibroblasts as an Inductor of Resistance to Photodynamic Therapy in Squamous Cell Carcinoma Cells. Cancers.

[2] Ruini C., Schlingmann S., Jonke Ž., Avci P., Padrón-Laso V., Neumeier F., Koveshazi I., Ikeliani I.U., Patzer K., Kunrad E. (2021). Machine Learning Based Prediction of Squamous Cell Carcinoma in Ex Vivo Confocal Laser Scanning Microscopy. Cancers.

[3] Curiel-Lewandrowski C., Myrdal C.N., Saboda K., Hu C., Arzberger E., Pellacani G., Legat F.J., Ulrich M., Hochfellner P., Oliviero M.C. (2021). In Vivo Reflectance Confocal Microscopy as a Response Monitoring Tool for Actinic Keratoses Undergoing Cryotherapy and Photodynamic Therapy. Cancers.

[4] Verzì A.E., Micali G., Lacarrubba F. (2021). Line-Field Confocal Optical Coherence Tomography May Enhance Monitoring of Superficial Basal Cell Carcinoma Treated with Imiquimod 5% Cream: A Pilot Study. Cancers.

[5] Giovannacci I., Meleti M., Garbarino F., Cesinaro A.M., Mataca E., Pedrazzi G., Reggiani C., Paganelli A., Truzzi A., Elia F. (2021). Correlation between Autofluorescence Intensity and Histopathological Features in Non-Melanoma Skin Cancer: An Ex Vivo Study. Cancers.

[6] Oya K., Nakamura Y., Zhenjie Z., Tanaka R., Okiyama N., Ichimura Y., Ishitsuka Y., Saito A., Kubota N., Watanabe R. (2021). Combination Treatment of Topical Imiquimod Plus Anti-PD-1 Antibody Exerts Significantly Potent Antitumor Effect. Cancers.

[7] Santiago J.L., Muñoz-Rodriguez J.R., Cruz-Morcillo M.A.d.l., Villar-Rodriguez C., Gonzalez-Lopez L., Aguado C., Nuncia-Cantarero M., Redondo-Calvo F.J., Perez-Ortiz J.M., Galan-Moya E.M. (2021). Characterization of Permeability Barrier Dysfunction in a Murine Model of Cutaneous Field Cancerization Following Chronic UV-B Irradiation: Implications for the Pathogenesis of Skin Cancer. Cancers.

[8] Ruini C., Schuh S., Gust C., Hartmann D., French L.E., Sattler E.C., Welzel J. (2021). In-Vivo LC-OCT Evaluation of the Downward Proliferation Pattern of Keratinocytes in Actinic Keratosis in Comparison with Histology: First Impressions from a Pilot Study. Cancers.

[9] Stanganelli I., Spagnolo F., Argenziano G., Ascierto P.A., Bassetto F., Bossi P., Donato V., Massi D., Massone C., Patuzzo R. (2022). The Multidisciplinary Management of Cutaneous Squamous Cell Carcinoma: A Comprehensive Review and Clinical Recommendations by a Panel of Experts. Cancers.

[10] Vlachos C., Tziortzioti C., Bassukas I.D. (2022). Paraneoplastic Syndromes in Patients with Keratinocyte Skin Cancer. Cancers.

[11] Omland S.H., Wenande E.C., Svane I.M., Tam J., Olesen U.H., Hædersdal M. (2021). Laser Immunotherapy: A Potential Treatment Modality for Keratinocyte Carcinoma. Cancers.

[12] Pihl C., Togsverd-Bo K., Andersen F., Haedersdal M., Bjerring P., Lerche C.M. (2021). Keratinocyte Carcinoma and Photoprevention: The Protective Actions of Repurposed Pharmaceuticals, Phytochemicals and Vitamins. Cancers.

[13] Romano A., Di Stasio D., Petruzzi M., Fiori F., Lajolo C., Santarelli A., Lucchese A., Serpico R., Contaldo M. (2021). Noninvasive Imaging Methods to Improve the Diagnosis of Oral Carcinoma and Its Precursors: State of the Art and Proposal of a Three-Step Diagnostic Process. Cancers.

[14] Caini S., Gnagnarella P., Stanganelli I., Bellerba F., Cocorocchio E., Queirolo P., Bendinelli B., Saieva C., Raimondi S., Gandini S. (2021). Vitamin D and the Risk of Non-Melanoma Skin Cancer: A Systematic Literature Review and Meta-Analysis on Behalf of the Italian Melanoma Intergroup. Cancers.

